# Sarcopenia is associated with hypertension in older adults: a systematic review and meta-analysis

**DOI:** 10.1186/s12877-020-01672-y

**Published:** 2020-08-06

**Authors:** Tingting Bai, Fang Fang, Feika Li, Yan Ren, Jiaan Hu, Jiumei Cao

**Affiliations:** Department of Geriatrics, Ruijin Hospital, Shanghai Jiaotong University School of Medicine, Shanghai, China

**Keywords:** Sarcopenia, Handgrip strength, Hypertension, Older adults, Meta-analysis

## Abstract

**Background:**

Sarcopenia, particularly low handgrip strength has been observed and correlated in association with hypertension among the older people. However, the results reported in different studies were inconsistent. In the current study, we conducted a systematic review and meta-analysis to reveal the significant association between sarcopenia, handgrip strength, and hypertension in older adults.

**Methods:**

PubMed, MEDLINE, Cochrane Library, and EMBASE databases were searched from inception to 15 November 2019 to retrieve the original research studies that addressed the association between sarcopenia, handgrip strength, and hypertension. All the relevant data were retrieved, analyzed, and summarized.

**Results:**

Twelve articles met the inclusion criteria and a total of 21,301 participants were included in the meta-analysis. Eight eligible studies have reported the odd ratios (ORs) of hypertension and sarcopenia, and the ORs ranged from 0.41 to 4.38. When pooled the ORs together, the summarized OR was 1.29 [95% confidence interval (CI) =1.00–1.67]. The summarized OR for the Asian group 1.50 (95% CI = 1.35–1.67) was significantly higher than that of Caucasian group 1.08 (95% CI = 0.39–2.97). Eleven studies have provided the data on association between handgrip strength and hypertension. The overall OR and 95% CI was 0.99 (95% CI = 0.80–1.23), showing no significant association.

**Conclusion:**

Sarcopenia was associated with hypertension, but no correlation was found between handgrip strength and hypertension in older adults.

## Background

The number of elderly people among the world population is expected to reach about 30% by the year 2050 [1]. However, the aging process is accompanied with alterations in some physiological systems leading to the development of geriatric syndromes and chronic diseases. It was previously reported that hypertension affects more than 70% of older people [2] and predisposes them to an increased risk of stroke (i.e., hemorrhagic and ischemic) and myocardial infarction [2, 3]. In the past few years, a large number of studies have indicated that hypertension is predominantly associated with elevated cardiovascular risk [4–6].

Recently, data from population studies have demonstrated that sarcopenia, a neuromuscular disease characterized by a progressive muscular atrophy accompanied by diminished muscle strength and/or lower muscle limb function, could be associated with with hypertension [7–9]. The consensus definitions of sarcopenia by European geriatric and gerontological societies proposed a mandatory measurement of handgrip strength to diagnose probable sarcopenia [10]. Meanwhile, sarcopenia has been demonstrated to have substantial associations with the aging process and can lead to significant morbidity and disability, including the loss of independence, poor quality of life, and mortality [11–14]. Sarcopenia has been reported to be associated with several contributing factors, such as primarily advanced age, immobility, inadequate nutrition, neurodegenerative disease, malignancy, chronic multiple endocrine disorders, and cardiometabolic disease [12]. Moreover, the rate of sarcopenia in the elderly is expected to increase in the future [15] and is becoming a major public health problem [16].

The handgrip strength examination is often applied as a diagnostic approach for sarcopenia in clinical settings, and such a measure is considered inexpensive, simple, easy, and can be implemented with a portable measuring tool. Until now, a significant association between sarcopenia and grip strength, and hypertension in older adults was controversial [7–9, 17, 18] and has not been systemically summarized. Therefore, a comprehensive investigation on this topic might allow an early detection of the key risk factors of hypertension in elderly patients suffering with sarcopenia and may help to effectively organize prevention or treatment strategies associated with specific vulnerability factors.

## Methods

### Literature search

A literature search was conducted using the individual and joint keywords, “handgrip strength”, “grip strength”, “sarcopenia”, and “hypertension” following the Preferred Reporting Items for Systematic Reviews and Meta-Analyses (PRISMA) statement [19]. To enhance the retrieval of potential literatures, we kept the search terms as broad as possible to identify the relevant publications. A systematic electronic data search of PubMed, MEDLINE, Cochrane Library, and EMBASE databases were performed up to 15 November 2019. Moreover, the bibliographies of all relevant studies and reviews, and Google Scholar for literature citing relevant studies were also checked and identified.

### Eligibility criteria

The inclusion criteria were as follows: (1) observational studies addressing the association between sarcopenia and hypertension, or handgrip strength and hypertension; (2) studies providing clear diagnostic criteria of sarcopenia and hypertension; (3) necessary data extracted from original studies; (4) studies published in English; and (5) only the study providing more detailed information was included if the population was reported in duplicate.

Reviews, case reports, abstracts or posters for conferences, studies focused on animal experiments or experiments in vitro, and studies published in languages other than English or Chinese were excluded.

### Data extraction

Two investigators (TTB and FF) have independently extracted the necessary information of included studies using a customized and standardized form, and the consensus were reached on all items by these two authors. For each included study, the following information were extracted: the author and year of publication, country, study design, sample size, patient demographic characteristics (e.g., age, sex, and nation), diagnostic criteria of sarcopenia and hypertension, sample size and characteristics for each group, follow-up period, and outcomes of each group.

### Quality scoring of studies

Two reviewers (FKL and JMC) have assessed the methodological strength of included studies independently in order to interpret the validity of any findings observed through the Newcastle-Ottawa Scale (NOS), a procedure performed to independently assess the methodological quality of meta-analysis of observational studies [20]. Newcastle-Ottawa Scale included three categories of factors: (1) patient selection (three items); (2) comparability of the two study arms (two items); and (3) assessment of the outcomes (two items). The detailed criteria for the three assessments are: whether the cases were defined adequately, the representativeness of the cases, the process of selection and definition of controls, comparability of cases and controls based on the design or analysis, ascertainment of exposure, the same method of ascertainment for cases and controls, and nonresponse rate.

Studies were awarded one star for each numbered item within the selection and exposure categories, and one or two stars for comparability. Studies were graded on an ordinal scoring scale. The scores ranged from 2 to 9 stars. Therefore, a scale of 0 to 4 stars was considered as poor quality, 5 to 6 stars as moderate quality, and 7 to 9 stars as high quality.

### Statistical analysis

The inverse variance method with random effects was conducted to summarize the dichotomous outcomes, odd ratios (ORs), and 95% confidence intervals (CIs). Stratified analyses were also performed with respect to the characteristics of the study population and outcome. Heterogeneity between included studies was assessed using the *I*^*2*^ and Q tests. Heterogeneity was defined as low, moderate, and high to I^2^ values of 25, 50, and 75%, respectively [21]. The Begg rank correlation [22] and Egger weighted regression methods [23] were used to assess the publication bias (*P* < 0.05 was considered indicative of a statistically significant publication bias). Review Manager (Version 5.3, The Cochrane Collaboration, Oxford, UK) was used for generation of forest plots and statistical analyses. The Begg and Egger tests were assessed by STATA 15.0 (Stata Corporation, College Station, TX, USA). A *P* value of < 0.05 was considered significant for all analysis.

## Results

### Study selection

**Fig. 1:**
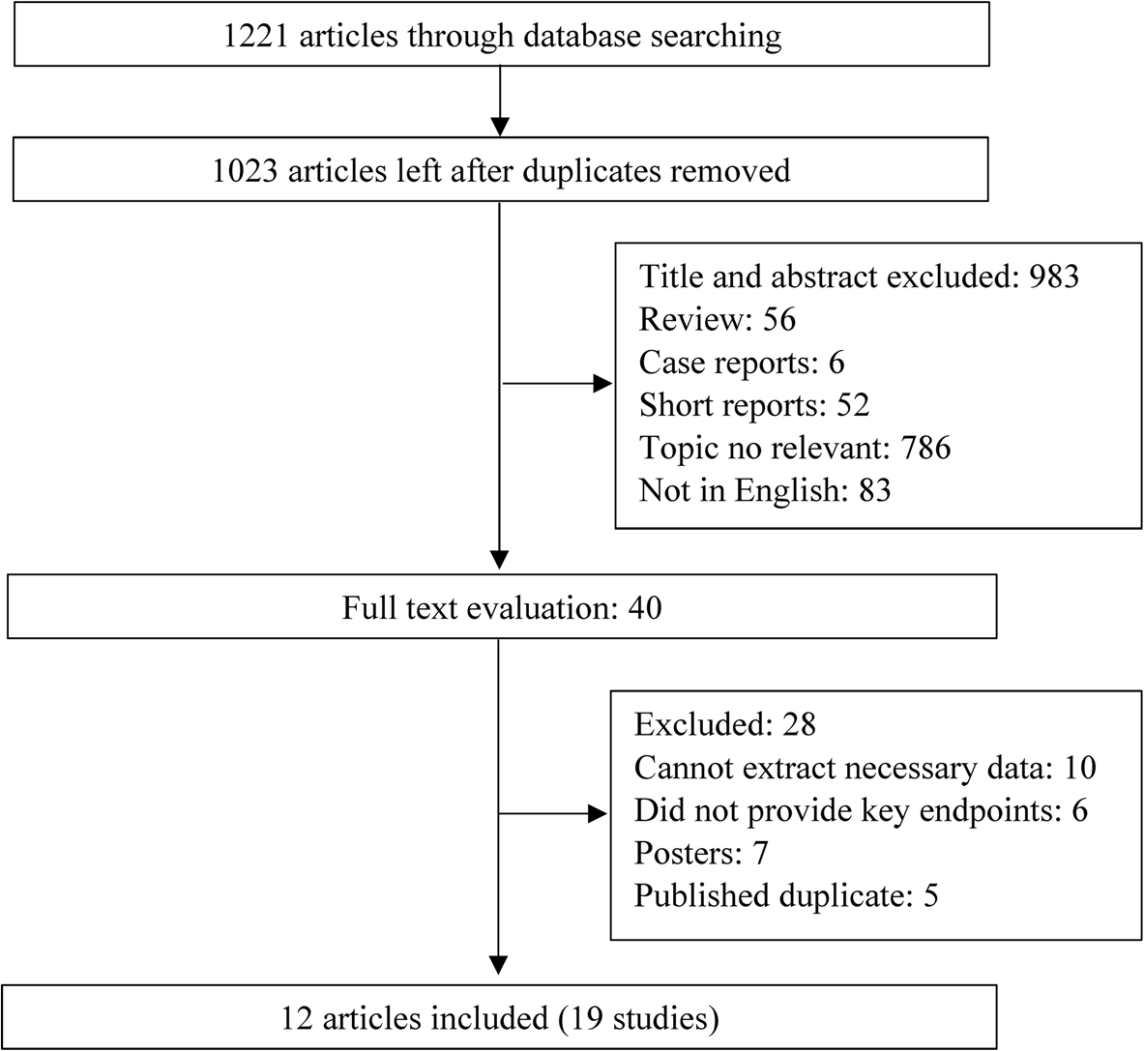
Flow chart of the study selection

In total, 1221 studies were retrieved as potentially relevant literature reports through the initial searches in different databases, after removing the duplicates, 1013 were left. The majority of potentially irrelevant literatures were excluded after reviewing the title or abstract. After retrieving 30 full-length manuscripts, finally, 12 articles [7–9, 17, 18, 24–30] of 19 studies were eligible for data extraction and meta-analysis. The flow chart of the studies enrolled in the current study can be found in Fig. 1. Of the 12 included articles, four articles [8, 17, 25, 30] reported the results according the characteristics of the included participants separately, such as by the sex and BMI. Therefore, during the processes of pooling the results from these articles, these articles were divided into two or more studies.

### Study characteristics

Twelve articles met the inclusion criteria and a total of 21,301 participants were included in the study. In the data analysis process, four articles [8, 16, 23, 28] were divided into two or four studies as the participants were categorized into several groups according their characteristics. Eight studies [7–9, 18, 27–29] addressed the association between sarcopenia and hypertension, and 11 studies [17, 24–26, 30] focused on the association between handgrip strength and hypertension. The sarcopenia was defined by three methods, the European Working Group on Sarcopenia in Older People (EWGSOP) criteria [31], and the Asian Working Group for Sarcopenia (AWGS) criteria. The hypertension was defined as systolic blood pressure (SBP) > 140 mmHg or diastolic blood pressure (DBP) > 90 mmHg. Part of the included studies defined handgrip strength following dynamometers according to the protocol from the Institute of Medicine [32]. The included studies were published between 2013 and 2019 and the sample size ranged from 72 to 4771. The participants’ demographic characteristics in the included studies can be found in Supplementary Table 1 and Supplementary Table 2.

Six studies were conducted in China [17, 18, 27, 30], two each in Republic of Korea [8] and Japan [25], and United States [9, 24, 33, 34], one each in Turkey [29], Switzerland [26], Italy [7], and Spain [28]. Most of the studies were cross-sectional studies except two cohort studies [7, 9]. The characteristics of the included studies and patients were summarized in Tables 1 and 2.

**Table 1:** Demographic and clinical characteristics of the subjects included in studies that focused on sarcopenia

Study included	No. of sarcopenia patients HTN (−)/HTN (+)	No. of non-sarcopenia patients HTN (−)/HTN (+)	Means ± SD (mmHg, non-sarcopenia/sarcopenia)		% of participants taking antihypertensive drugs	Odd ratios (95% CI) ^c^
			SBP	DBP		
Landi et al. [7]	154/126	43/28				
Han et al. ^a^ [8]	2326/1156	894/544	126.40 ± 0.50/129.30 ± 0.90	74.30 ± 0.30/75.10 ± 0.50	33.00 ± 1.30/ 45.30 ± 1.90	1.50 (1.23–1.84)
Han et al. ^b^ [8]	594/393	1032/771	129.90 ± 0.80/131.20 ± 0.70	76.90 ± 0.50/77.60 ± .40	52.00 ± 2.40/ 62.30 ± 1.90	NA
Koo et al. [9]	239/98	70/41	98.10 ± 14.8/ 127.9 ± 17.4	77.8 ± 11.0/ 77.2 ± 11.5	NA	NA
Can et al. [29]	36/4	36/12	NA	NA	NA	NA
Han et al. [24]	634/267	77/36	NA	NA	NA	NA
Montes et al. [28]	148/116	52/33	NA	NA	NA	NA
Xu et al. [18]	4459/NA	312/NA	NA	NA	NA	1.44 (1.16–1.78)

*Abbreviations*: *HTN* Hypertension, *SD* standard deviation, *SBP* systolic blood pressure, *DBP* diastolic blood pressure, *CI* confidence interval, *NA* not available

^a^_,_ participants whose BMI was less than 25.00 kg/m^2^

^b^_,_ participants whose BMI was equal or more than 25.00 kg/m^2^

^c^_,_ Odd ratios stands for hypertension with sarcopenia compared to without sarcopenia

**Table 2:** Demographic characteristics of the subjects included in studies that focused on handgrip strength

Study included	Sample size	Handgrip strength (means ± SD, per kg)	Odd ratios (95% CI) for High blood pressure ^g^	β of linear regression (standard error)
Mainous, et.al., 2015 [24]	1469	60.8 ± 1.61 ^a^/71.5 ± 0.84 ^b^	NA	−4.93 (0.03)
Kawamoto, et.al., 2016 ^a^ [25]	742	33.40 ± 7.50	0.78 (0.66, 0.94)	NA
Kawamoto, et.al., 2016 ^b^ [25]	937	21.30 ± 4.10	0.72 (0.62, 0.83)	NA
Gubelmann, et.al., 2017 ^a^ [26]	1891	NA	1.23 (1.04–1.46)	NA
Gubelmann, et.al., 2017 ^b^ [26]	1577	NA	1.01 (0.80–1.27)	NA
Ji, et.al., 2018 ^a^ [17]	2184	41.50 ± 8.80	1.23 (1.04, 1.46)	0.21 (0.09)
Ji, et.al., 2018 ^b^ [17]	2413	26.70 ± 5.70	1.01 (0.80, 1.27)	0.01 (0.12)
Ji, et.al., 2018 ^c^ [17]	563	NA	1.14 (0.83, 1.55)	0.13 (0.16)
Ji, et.al., 2018 ^d^ [17]	1292	NA	1.29 (1.04, 1.59)	0.030 (0.17)
Ji, et.al., 2018 d ^e^ [17]	636	NA	0.88 (0.58, 1.33)	−0.13 (0.21)
Ji, et.al., 2018 d ^f^ [17]	1323	NA	1.02 (0.76, 1.38)	0.02 (0.15)
Zhang, et.al., 2019 ^a^ [30]	515	35.94 ± 19.72	0.55 (0.28, 1.08)	NA
Zhang, et.al., 2019 ^b^ [30]	637	14.45 ± 10.41	0.19 (0.07, 0.55)	NA

*Abbreviations*: *CI* confidence interval, *SD* Standard deviation, *NA* not available

^a^, males

^b^, females

^c^, Underweight or normal body mass index of males

^d^, Underweight or normal body mass index of females

^e^, Overweight body mass index of males

^f^, Overweight or obese body mass index of females

^g^, Odd ratios stands for hypertension with sarcopenia compared to without sarcopenia

### Quality assessment of studies

Newcastle-Ottawa Scales for the eligible studies were presented in Supplementary Table 3 and all included studies are found to exhibit an acceptable quality. Four studies were evaluated as 6 stars, 6 studies were 7 stars, and 2 studies were 8 stars.

### The association between sarcopenia and hypertension

**Fig. 2:**
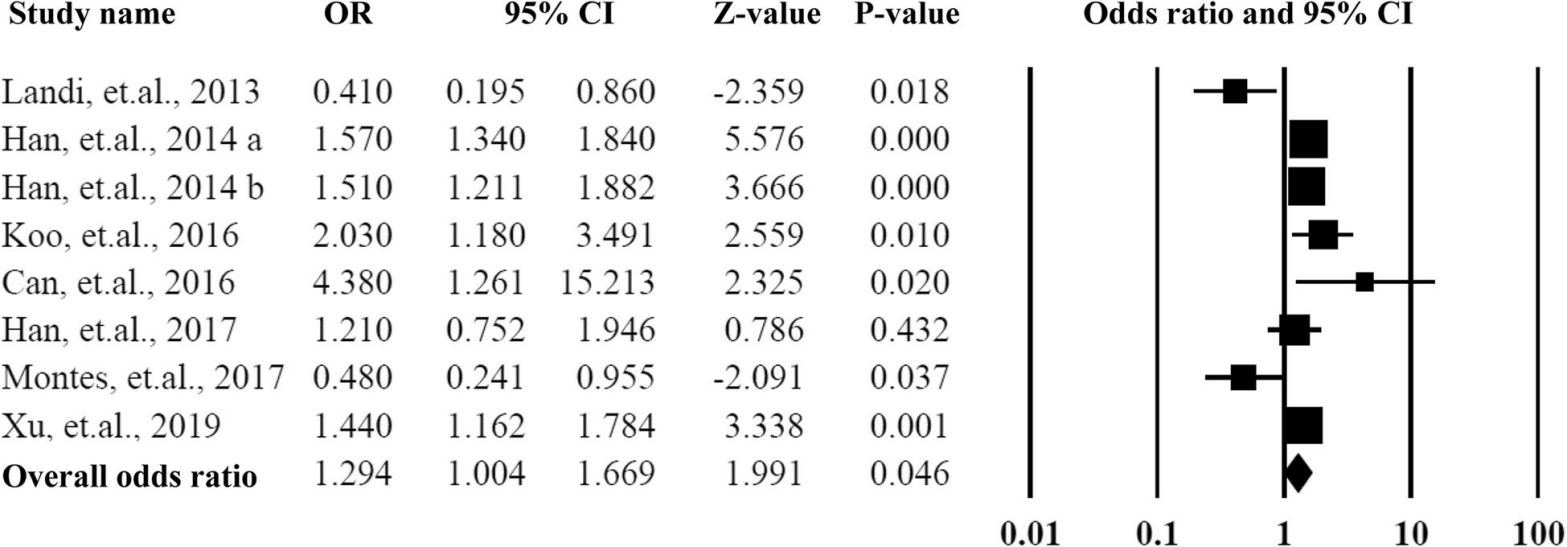
Summarized overall odds ratio of hypertension among sarcopenia and non- sarcopenia patients

All the eight eligible studies have reported the ORs of hypertension, and the ORs ranged from 0.41 to 4.38. When pooled the ORs together, the summarized OR was 1.29 (95% CI = 1.00–1.67, *P* = 0.04) with a moderate heterogeneity (*I*^*2*^ = 74%). The detailed information could be found in Fig. 2 and Supplementary Fig. 1.

**Fig. 3:**
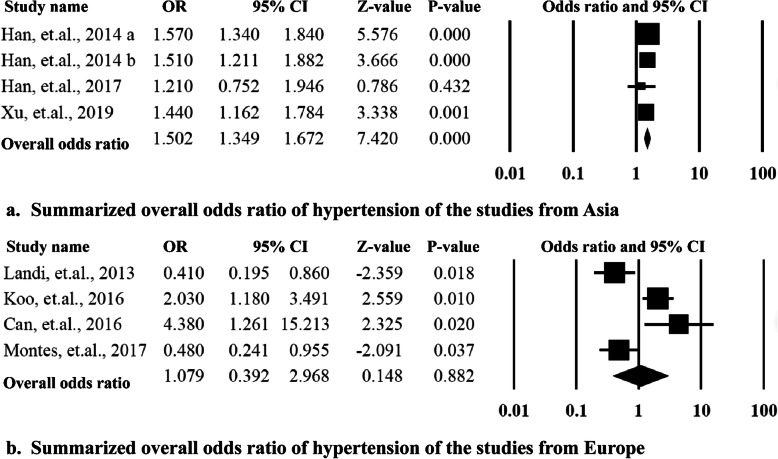
Summarized overall odds ratio of hypertension among the studies grouped by ethnicity of the participants

**Fig. 4:**
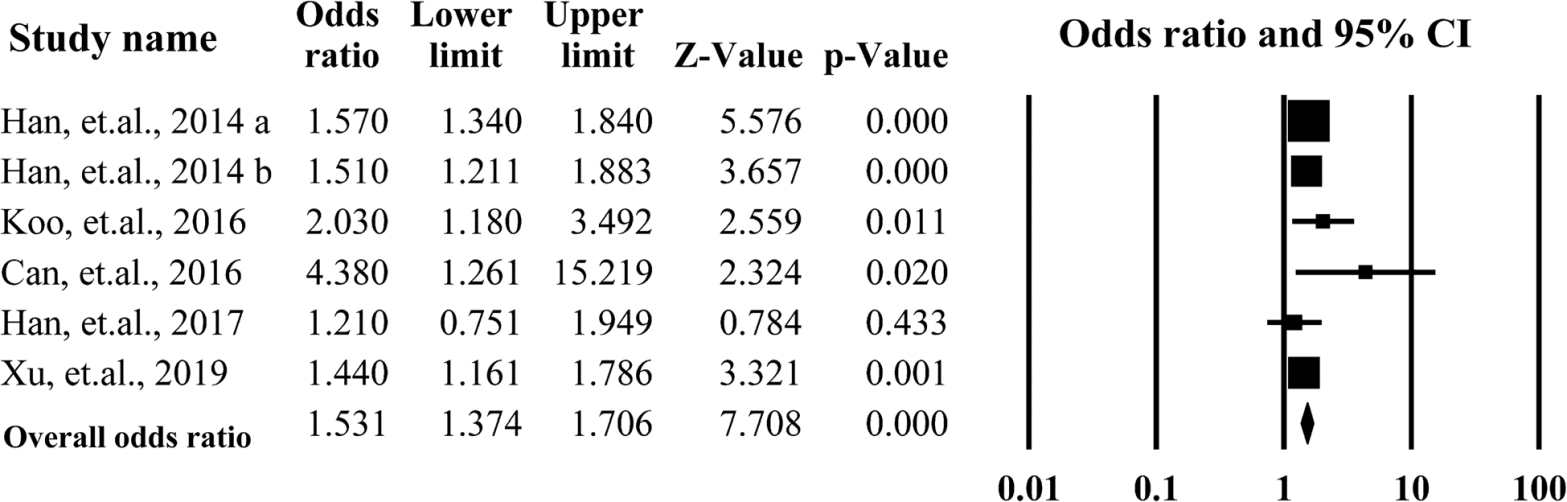
Summarized overall odds ratio of hypertension among the studies with equal to or more than 7 stars according the Newcastle-Ottawa Scales

To explore the sources of heterogeneity, the subgroup analysis was performed by categorizing the studies according to the ethnicity of the participants and the Newcastle-Ottawa Scales than were equal to or more than 7 stars. The Asian group included 4 studies from China and Korea, the Caucasian group included four studies conducted in United States, Italy, Spain, and Turkey. The summarized OR for the Asian group 1.50 (95% CI = 1.35–1.67, *P* = 0.00) was significantly higher than that of the Caucasian group 1.08 (95% CI = 0.39–2.97, *P* = 0.88). The heterogeneities for the two subgroups were significantly decreased to *I*^2^ = 34% and *I*^2^ = 40%. When the studies with low quality were removed (Newcastle-Ottawa Scales< 6), the overall OR was 1.53 (95%CI = 1.37–1.71, *P* = 0.00) with lower heterogeneity (*I*^2^ = 2.62%). More data is presented in Figs. 3 and 4.

### The association between handgrip strength and hypertension

**Fig. 5:**
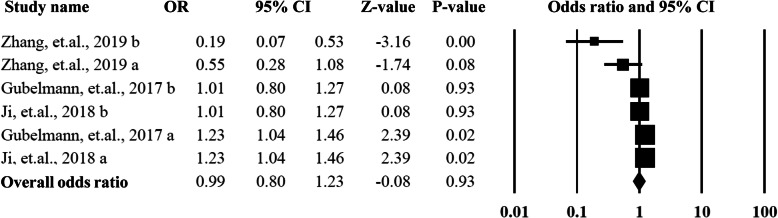
Summarized overall odds ratio of handgrip strength among hypertension and non-hypertension patients

Eleven studies provided data on association between handgrip strength and hypertension, and among them 10 studies reported the odds ratios and 95% CI. The overall OR and 95% CI was 0.99 (95% CI = 0.80–1.23, *P* = 0.93) with a higher heterogeneity (*I*^2^ = 76%) and significant publication bias (*P* < 0.01). The detailed data can be found in Fig. 5.

**Fig. 6:**
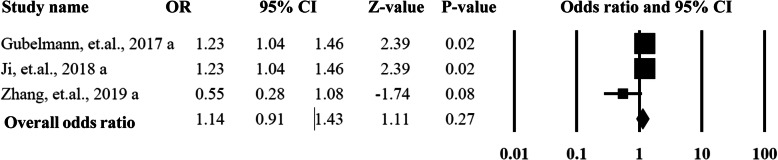
Summarized overall odds ratio of handgrip strength based on male participants among hypertension and non-hypertension patients

**Fig. 7:**
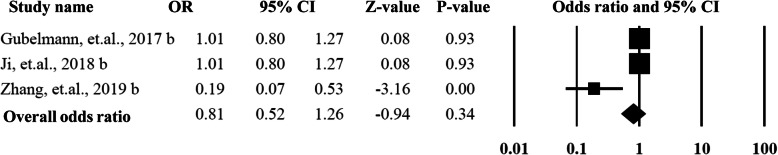
Summarized overall odds ratio of handgrip strength based on female participants among hypertension and non-hypertension patients

As shown in Figs. 6 and 7, in order to explore the sources of heterogeneity and publication bias, the included studies were categorized into two groups by the gender of the participants. For the males, the pooled OR was 1.14 (95%CI = 0.91–1.43, *P* = 0.27) with an acceptable heterogeneity (*I*^2^ = 31%) and without publication bias (*P* > 0.05). The female group did not show any statistically significant difference with an OR as 0.81 (95%CI = 0.52–1.26, *P* = 0.34, *I*^2^ = 45%) without publication bias (*P* > 0.05).

**Fig. 8:**
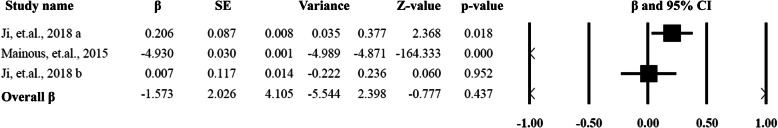
Summarized overall β for the linear regression and standard error

Seven studies have reported the β value and standard error of the linear regression on hypertension and the pooled β value was − 1.57 with an SE equal to 1.03 (Fig. 8), and the heterogeneity was 99%. As two studies have provided the data on different body mass indexes, two more subgroup analysis were done, i.e. underweight or normal body mass index group (OR = 1.04, 95%CI = 0.81–1.33, *P* = 0.77), and overweight or obese body mass index group (OR = 1.18, 95%CI = 0.94–1.41, *P* = 0.16). The data are presented in Supplementary Fig. 3 and Supplementary Fig. 4.

### Publication bias

Most of the analysis except one has shown potential publication bias among the included trials according to Begg rank correlation analysis and Egger weighted regression analysis (*P* value of the analysis was more than 0.05). The detailed potential publication bias of each analysis can be found in Supplementary Table 4.

## Discussion

To the best of our knowledge, the current meta-analysis is the first systematic review and meta-analysis study summarizing the association between sarcopenia and hypertension, and the association of handgrip strength and hypertension. Nineteen studies with 21,301 participants were included, among which eight studies have addressed the association between sarcopenia and hypertension and indicated that sarcopenia was a risk factor for the hypertension. Eleven studies have focused on the association between handgrip strength and hypertension and no association was found by the pooled results.

In the current study, sarcopenia showed an association with hypertension. Several prospective and cross-sectional studies have found a correlation between sarcopenia and hypertension [8, 18]. In the current study, Asian Working Group for Sarcopenia (AWGS) criteria and the European Working Group on Sarcopenia in Older People (EWGSOP) criteria were used, which showed significantly different ORs. This might partly explain why Asian groups had a stronger association with hypertension than that of Caucasian group. Many potential mechanisms for sarcopenia have been researched, such as chronic inflammation [35], and its relevant catabolic cytokines remain the most widely accepted mechanism of sarcopenia [35]. It was also reported that chronic inflammation and its relevant catabolic cytokines production is the major risk factor for age-related chronic diseases, such as hypertension [8]. Sarcopenia is not causing hypertension, but it may increase the likelihood of having the other through this shared mechanism and sarcopenia now needs to be recognized in routine clinical settings.

In the current study, handgrip strength was significantly associated with hypertension in both men and women, and these results were controversial in various other studies [25, 26]. However, regular exercise, which has been shown consistently in plenty of studies to improve blood pressure, may eventually improve the mitochondrial function, reduce inflammation, enhance metabolic functions and alleviate sarcopenia symptoms [36].

Frailty is the most problematic expression of population ageing. It is a state of vulnerability to poor resolution of homeostasis and is a consequence of cumulative decline in many physiological systems during a lifetime. The adverse outcomes of frailty might be mediated by sarcopenia, which may be considered the biological substrate for the development of frailty and the related negative health outcomes [37]. Most importantly, it is becoming exceedingly important for us to verify whether preventive strategies focusing on the early detection and treatment of sarcopenia could lead to better survival in the older people.

It is necessary to consider the limitations of the present meta-analysis while interpreting the results. First, the definition of sarcopenia was inconsistent in different studies and the variations in assessment of sarcopenia across studies could have caused methodological limitations and compromised the results. Second, the number of the included studies was limited and the majority of them were from Asian countries. As sarcopenia might be affected by the economic level, medical level, and genetic factors, the associations between sarcopenia, handgrip strength, and hypertension in different countries could be different. Therefore, the result in the current study can only partly annotate the associations. Third, almost all of the studies addressing sarcopenia did not provide the specific breakdown of sarcopenia measurements by gender and age. Due to the limited sample size and information in each study we could not perform more subgroup or sensitivity analyses, e.g. the sensitivity analyses based on the age and sex. Moreover, due to the large disparity in the numbers of participants among the included studies, the conclusions related to the association of sarcopenia with hypertension might be overstated. Four, potential language bias might have existed because our literature searches only considered articles published in English or Chinese.

## Conclusions

In conclusion, our meta-analysis provided pooled results based on 19 studies from eight different countries and summarized a large data set of 21,301 participants. The current study highlighted that sarcopenia was significantly associated with hypertension. In the future, by stratifying patients, efforts must be made to prevent and treat sarcopenia in the older population. At the same time, performing larger sample size studies from different countries in the future may substantially corroborate the conclusive remarks derived from this study.

## Supplementary information

**Additional file 1: Supplementary Figure 1.** Funnel plot for the overall odds ratio of hypertension among sarcopenia and non- sarcopenia patients. **Supplementary Figure 2.** Funnel plot for the odds ratio of handgrip strength among hypertension and non-hypertension patients. **Supplementary Figure 3.** Summarized overall odds ratio of underweight or normal body mass index (BMI) in patients. **Supplementary Figure 4.** Summarized overall odds ratio of overweight or obese body mass index in patients.

**Additional file 2: Supplementary Table 1.** Demographic and clinical characteristics of the subjects included in studies that focused on sarcopenia. **Supplementary Table 2.** Demographic and clinical characteristics of the subjects included in studies that focused on handgrip strength. **Supplementary Table 3.** Quality assessment of included studies by Newcastle-Ottawa Scale. **Supplementary Table 4.** Publication bias of summarized outcome.

**Additional file 3.** Search strategy.

## Data Availability

Since it is a meta-analysis, all data were extracted from public database, then all data were available.

## Abbreviations

ORs: Odd ratios
CI: Confidence interval
PRISMA: Preferred Reporting Items for Systematic Reviews and Meta-Analyses
NOS: Newcastle-Ottawa Scale
AWGS: Asian Working Group for Sarcopenia
EWGSOP: European Working Group on Sarcopenia in Older People
