# Supplementary material for: Sarcopenia is associated with hypertension in older adults: a systematic review and meta-analysis
Source: BMC Geriatr. 2020 Aug 6;20:279. doi: 10.1186/s12877-020-01672-y (PMC7409686; doi:10.1186/s12877-020-01672-y)
Supplement: Supplementary file 1 — Additional file 1: Supplementary Figure 1. Funnel plot for the overall odds ratio of hypertension among sarcopenia and non- sarcopenia patients. Supplementary Figure 2. Funnel plot for the odds ratio of handgrip strength among hypertension and non-hypertension patients. Supplementary Figure 3. Summarized overall odds ratio of underweight or normal body mass index (BMI) in patients. Supplementary Figure 4. Summarized overall odds ratio of overweight or obese body mass index in patients. [file 12877_2020_1672_MOESM1_ESM.docx]

Supplementary Figure 1. Funnel plot for the overall odds ratio of hypertension in patients.





Supplementary Figure 2. Funnel plot for the odds ratio of handgrip strength in patients.


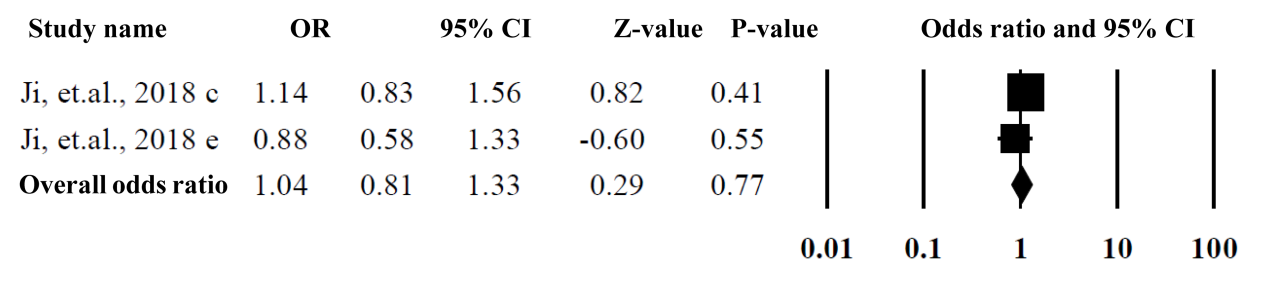


Supplementary Figure 3. Summarized overall odds ratio of underweight or normal body mass index (BMI) in patients.


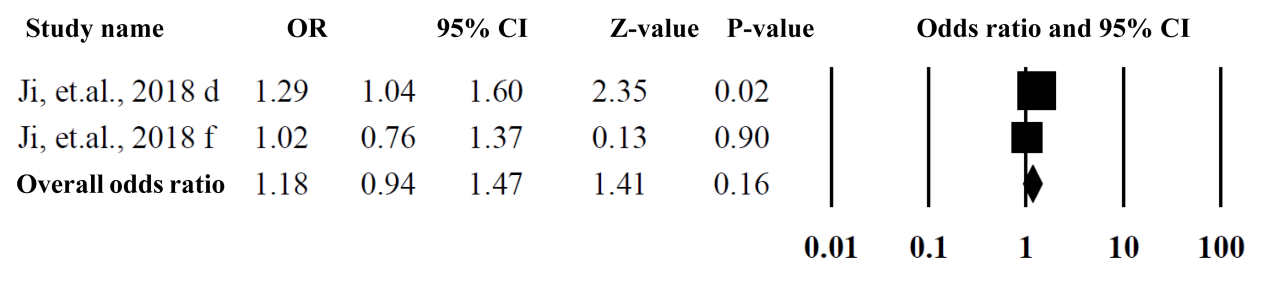


Supplementary Figure 4. Summarized overall odds ratio of overweight or obese body mass index in patients.
