# Supplementary material for: Sarcopenia is associated with hypertension in older adults: a systematic review and meta-analysis
Source: BMC Geriatr. 2020 Aug 6;20:279. doi: 10.1186/s12877-020-01672-y (PMC7409686; doi:10.1186/s12877-020-01672-y)
Supplement: Supplementary file 2 — Additional file 2: Supplementary Table 1. Demographic and clinical characteristics of the subjects included in studies that focused on sarcopenia. Supplementary Table 2. Demographic and clinical characteristics of the subjects included in studies that focused on handgrip strength. Supplementary Table 3. Quality assessment of included studies by Newcastle-Ottawa Scale. Supplementary Table 4. Publication bias of summarized outcome. [file 12877_2020_1672_MOESM2_ESM.docx]

**Supplementary Table 1. Demographic and clinical characteristics of subjects included in studies that focused on sarcopenia**

| **Study included** | **Country** | **Study design** | **Gender (% of males)** | | **Age, years (means±SD)** | | **Diagnostic criteria** | |
| --- | --- | --- | --- | --- | --- | --- | --- | --- |
|  |  |  | **sarcopenia (-)** | **sarcopenia (+)** | **sarcopenia (-)** | **sarcopenia (+)** | **Sarcopenia** | **HTN** |
| Landi*, et.al*., 2013 | Italy | Prospective cohort | 39.53 | 31.81 | 82.20 ± 1.40 | 82.20 ± 1.30 | EWGSOP | SBP>140 mmHg or DBP>90 mmHg ^c^ |
| Han*, et.al.*, 2014 ^a^ | Korea | Cross-sectional | 44.80 | 59.30 | 69.80±0.20 | 70.60±0.30 | ASM/weight  (kg) less than 1 SD | SBP>140 mmHg or DBP>90 mmHg ^c^ |
| Han, *et.al.*, 2014 ^b^ | Korea | Cross-sectional | 27.10 | 31.60 | 71.40±0.40 | 72.90±0.40 | ASM/weight  (kg) less than 1 SD | SBP>140 mmHg or DBP>90 mmHg ^c^ |
| Koo, *et.al*., 2016 | United States | Cross-sectional cohort | 47.70 | 44.30 | 52.5 0± 13.20 | 54.9 ± 17.0 | ASM/weight  (kg) less than 2 SD | SBP>140 mmHg or DBP>90 mmHg ^c^ |
| Can, *et.al.*, 2016 | Turkey | Cross-sectional | 33.33 | 41.67 | 77.30 ± 6.20 | 80.20 ± 6.30 | EWGSOP ^d^ | SBP>140 mmHg or DBP>90 mmHg ^c^ |
| Xu, *et.al.*, 2019 | China | Cross-sectional | 37.81 | NA | 61.40 (11.35)/ 59.10 (11.33) | NA | AWGS ^e^ | medical history or SBP>140 mmHg or DBP>90 mmHg |
| Han, *et.al.*, 2017 | China | Cross-sectional | 50.50 | 37.70 | 66.33 ± 5.52 | 72.71 ± 7.58 | AWGS | SBP>140 mmHg or DBP>90 mmHg ^c^ |
| Montes, *et.al*., 2017 | Spain | Cross-sectional | 86.90 | 13.10 | 76.20 ± 0.40 | 78.10 ± 0.70 | EWGSOP | SBP>140 mmHg or DBP>90 mmHg ^c^ |

Abbreviations: HTN, Hypertension; SD, standard deviation; ASM, appendicular skeletal muscle mass; NA, not available.

^a^_,_ participants whose BMI was less than 25.00 kg/m^2^.

^b^_,_ participants whose BMI was equal or more than 25.00 kg/m^2^.

^c^_,_ subjects taking antihypertensive medications, systolic blood pressure greater than 140 mmHg, or diastolic blood pressure greater than 90 mmHg.

^d^, European Working Group on Sarcopenia in Older People (EWGSOP) criteria.

^e^, Asian Working Group for Sarcopenia (AWGS) criteria.

**Supplementary Table 2. Study participants’ characteristics of the included studies** **that focused on** **handgrip strength**

| **Study included** | **Country** | **Study design** | **Gender (% of Males)** | **Age, years (means±SD)** | **Diagnostic criteria** | | **Means±SD** | |
| --- | --- | --- | --- | --- | --- | --- | --- | --- |
|  |  |  |  |  | **Handgrip strength** | **HTN** | **SBP** | **DBP** |
| Mainous, et.al., 2015 | United States | Cross-sectional | 44.80 | Age 20-64 85.7 %  Age >65 14.30 % ^d^ | Takei digital grip strength dynamometer | SBP>140 mmHg or DBP>90 mmHg ^a^ | NA | NA |
| Kawamoto, et.al., 2016 ^c^ | Japan | Cross-sectional | 100.00 | 70.00±9.00 | NA | SBP>140 mmHg or DBP>90 mmHg ^a^ | NA | NA |
| Kawamoto, et.al., 2016 ^d^ | Japan | Cross-sectional | 0.00 | 70.00±8.00 | Unknown Dynamometer | SBP>140 mmHg or DBP>90 mmHg ^a^ | NA | NA |
| Gubelmann, et.al., 2017 | Switzerland | Cross-sectional | 42.00 | 69.32±6.32 | Unknown Dynamometer | SBP>140 mmHg or DBP>90 mmHg ^a^ | NA | NA |
| Ji, et.al., 2018 ^c^ | China | Cross-sectional | 100.00 | 47.10 ± 18.30 | Takei Grip-D dynamometer | SBP>140 mmHg or DBP>90 mmHg ^a^ | 124.50 ± 16.30 | 70.80 ± 12.00 |
| Ji, et.al., 2018 ^d^ | China | Cross-sectional | 0.00 | 47.80 ± 18.30 | Takei Grip-D dynamometer | SBP>140 mmHg or DBP>90 mmHg ^a^ | 121.20 ± 18.8 | 68.80 ± 10.80 |
| Zhang, et.al., 2019 ^b^ | China | Cross-sectional | 100.00 | 63.71 ± 9.36 | Takei Grip-D dynamometer | SBP>140 mmHg or DBP>90 mmHg | 128.70 ± 13.84 | 79.99 ± 8.20 |
| Zhang, et.al., 2019 ^c^ | China | Cross-sectional | 0.00 | 65.83 ± 10.21 | Takei Grip-D dynamometer | SBP>140 mmHg or DBP>90 mmHg | 128.26 ±14.84 | 78.48 ± 8.54 |

Abbreviations: HTN, Hypertension; SD, standard deviation; SBP, systolic blood pressure; DBP, diastolic blood pressure; NA, not available.

^a^_,_ subjects taking antihypertensive medications, systolic blood pressure greater than 140 mmHg, or diastolic blood pressure greater than 90 mmHg.

^b^, only included males.

^c^, only included females.

^d^, percentage of the participants younger and older than 65 years.

**Supplementary Table 3. Quality assessment of included studies by Newcastle-Ottawa Scale ^a^**

| **Study included** | **Overall quality score** |
| --- | --- |
| Landi, et.al., 2013 | 6 |
| Han, et.al., 2014 | 7 |
| Koo, et.al., 2016 | 7 |
| Can, et.al., 2016 | 7 |
| Xu, et.al., 2019 | 7 |
| Han, et.al., 2017 | 7 |
| Montes, et.al., 2017 | 6 |
| Mainous, et.al., 2015 | 7 |
| Kawamoto, et.al., 2016 | 8 |
| Gubelmann, et.al., 2017 | 7 |
| Ji, et.al., 2018 | 8 |
| Zhang, et.al., 2019 | 7 |

^a^, The study quality was assessed according to the Newcastle Ottawa Quality assessment scale for cross-sectional studies or cohort studies. This scale awards a maximum of 9 points to each study.

**Supplementary Table 4. Publication bias of summarized outcomes**

| **Outcomes** | **Begg (*P* value)** | **Egger (*P* value)** |
| --- | --- | --- |
| Summarized overall odds ratio of hypertension | 0.45 | 0.34 |
| Summarized overall odds ratio of hypertension among the studies from Asia | 0.16 | 0.10 |
| Summarized overall odds ratio of hypertension among the studies from Europe | 0.21 | 0.12 |
| Summarized overall odds ratio of handgrip strength | 0.01 | 0.01 |
| Summarized overall odds ratio of handgrip strength based on male participants | 0.60 | 0.15 |
| Summarized overall odds ratio of handgrip strength based on female participants | 0.33 | 0.20 |
| Summarized overall β for the linear regression and standard error | 0.54 | 0.31 |
