# Supplementary material for: Sarcopenia is associated with hypertension in older adults: a systematic review and meta-analysis
Source: BMC Geriatr. 2020 Aug 6;20:279. doi: 10.1186/s12877-020-01672-y (PMC7409686; doi:10.1186/s12877-020-01672-y)
Supplement: Supplementary file 3 — Additional file 3. Search strategy. [file 12877_2020_1672_MOESM3_ESM.docx]

**Sarcopenic is associated with hypertension in older adults: a systematic review and meta-analysis**

1#

(Handgrip Strength) OR (grip strength)

2#

Sarcopenia

3#

Hypertension OR(high blood pressure) OR (Hypertensive diseases)

Combine: (1# OR 2#) AND 3#
